# A Novel Small-Molecule TLR7 Agonist AXC-715 Stabilizes TLR7 Dimerization and Exhibits Broad-Spectrum Antiviral Activity

**DOI:** 10.3390/microorganisms14040862

**Published:** 2026-04-11

**Authors:** Chen Yao, Meng-Hua Du, Yan-Jie Ma, Heng Wang, Liu Hang, Zhi-Cheng Li, Hong-Yu Yang, Guo-Yu Yang, Meng-Di Wang, Sheng-Li Ming

**Affiliations:** 1 College of Veterinary Medicine, Henan Agricultural University, Zhengzhou 450046, China; 2 Key Laboratory of Animal Biochemistry and Nutrition, Ministry of Agriculture and Rural Affairs, Zhengzhou 450046, China; 3 College of Life Sciences, Henan University of Animal Husbandry and Economy, Zhengzhou 450046, China; 4 Key Laboratory of Veterinary Biotechnology of Henan Province, Henan Agricultural University, Zhengzhou 450046, China

**Keywords:** AXC-715, TLR7, dimerization, NF-κB activation, antiviral activity, pseudorabies virus, vesicular stomatitis virus

## Abstract

Toll-like receptor 7 (TLR7) agonism offers a promising avenue for antiviral intervention. This study characterizes AXC-715, a novel small-molecule agonist that selectively targets TLR7 to elicit broad-spectrum antiviral effects. Structural analysis of the AXC-715–hTLR7 complex (PDB ID: 5GMH) elucidates the molecular basis of receptor activation. AXC-715 occupies the interface of TLR7 monomers, establishing critical hydrogen bonds with D555 and T586, alongside π-π and π-alkyl interactions with F408, V381, and L557. These interactions effectively promote and stabilize the active TLR7 dimeric conformation. Functionally, AXC-715 activates NF-κB signaling in a P65-dependent manner without inducing cytotoxicity in PK-15 or THP-1 cells. In vitro assays demonstrated that AXC-715 potently inhibits the replication of both pseudorabies virus (PRV) and vesicular stomatitis virus (VSV) by specifically impairing viral replication, distinct from adsorption, entry, assembly, or release processes. The antiviral effect was abolished in TLR7-knockout PK-15 cells, confirming the strict dependence of AXC-715 on on-target TLR7 signaling. These findings highlight AXC-715 as a potent TLR7 agonist that stabilizes receptor dimerization to inhibit viral replication, providing a valuable framework for developing TLR7-based antiviral therapeutics.

## 1. Introduction

Innate immune defense against viral pathogens relies on pattern recognition receptors (PRRs) that detect conserved pathogen-associated molecular patterns (PAMPs) and initiate host defense programs [1,2]. Among these, Toll-like receptor 7 (TLR7) is localized within endosomal compartments, where it senses viral single-stranded RNA (ssRNA). Upon ligand recognition, TLR7 triggers MyD88-dependent signaling cascades, leading to the activation of nuclear factor-κB (NF-κB) and interferon regulatory factor 7 (IRF7). This activation results in the induction of type I interferons (IFN-I) and proinflammatory cytokines, which constitute a critical axis of antiviral innate immunity [3,4,5,6]. Dysregulated TLR7 signaling is associated with impaired viral clearance and excessive inflammatory responses [7], underscoring the necessity for precise modulation of TLR7 activity to balance antiviral protection with immunopathology.

Pharmacological activation of TLR7 has emerged as a viable strategy to enhance host antiviral immunity [8,9,10]. Early investigations demonstrated that small-molecule compounds could activate immune responses via TLR7-dependent pathways, exerting potent antiviral effects [11]. Subsequently, multiple TLR7 agonists, including imiquimod and its derivatives, have been developed for antiviral and immunotherapeutic applications [12,13,14]. Recent advances in medicinal chemistry have facilitated the discovery of novel small-molecule TLR7 agonists with improved potency and pharmacological profiles [15]. However, challenges persist, including limited receptor selectivity and an incomplete understanding of the mechanisms governing broad-spectrum efficacy. Whether pharmacological TLR7 activation can confer effective protection against both DNA and RNA viruses remains insufficiently explored [16]. Thus, the identification of structurally defined, functionally potent TLR7 agonists with broad-spectrum activity addresses a critical unmet need [17,18].

Structural biology has provided profound insights into the molecular mechanisms of TLR7 activation. Crystallographic and cryo-electron microscopy studies have revealed that TLR7 forms an active dimer upon ligand binding [19,20], with both guanosine and ssRNA contributing to activation through coordinated interactions within the ligand-binding pocket [20,21]. These findings established the structural basis for ligand-induced dimerization and signal initiation, offering a rational framework for structure-guided drug design. Nevertheless, relatively few small molecules have been structurally characterized in complex with TLR7, and the precise conformational mechanisms governing agonist-induced activation require further elucidation.

Pseudorabies virus (PRV), a neurotropic double-stranded DNA alphaherpesvirus, is the causative agent of Aujeszky’s disease and poses a significant threat to the global swine industry. PRV has evolved multiple immune evasion strategies that antagonize host innate immune signaling, particularly type I interferon responses [22], thereby facilitating efficient replication and persistence [23,24]. Conversely, vesicular stomatitis virus (VSV), a negative-strand RNA virus, is highly sensitive to interferon-mediated restriction and serves as a robust model for investigating innate antiviral immunity [25,26]. Given their distinct genomic architectures, replication strategies, and immune susceptibilities, PRV and VSV represent complementary models for evaluating antiviral mechanisms and the breadth of therapeutic efficacy.

This study reports the identification and characterization of AXC-715, a novel small-molecule TLR7 agonist. Structural analyses reveal that AXC-715 binds at the TLR7 monomer interface, stabilizing the active dimeric conformation through coordinated hydrogen-bonding and hydrophobic interactions. Functionally, AXC-715 robustly activates NF-κB signaling without detectable cytotoxicity. Using PRV and VSV infection models, we demonstrate that AXC-715 markedly suppresses viral replication in vitro, exhibiting broad-spectrum activity against both DNA and RNA viruses. Mechanistic studies confirm that the antiviral effects are strictly dependent on TLR7 signaling, defining its specific mode of action. These results establish AXC-715 as a structurally defined, functionally potent TLR7 agonist, providing mechanistic insights into TLR7-targeted antiviral immunomodulation.

## 2. Materials and Methods

### 2.1. General Information on Chemistry

All solvents and reagents were commercially available and used without further purification unless otherwise stated. 1^1^H NMR and 13^13^C NMR spectra were acquired in CDCl_3_, D_2_O or DMSO-d_6_ using a Bruker DPX-400 Spectrometer (Rheinstetten, Germany), with data processed by MestReNova 14.0.23239 (Santiago de Compostela, Spain) software. In 13^13^C NMR spectra, the middle signal of CDCl_3_ was referenced to 77.16 ppm. Chemical shifts are reported in δ units (ppm) relative to an internal standard. Signal multiplicity is reported as singlet (s), doublet (d), triplet (t), quartet (q), and multiplet (m). Coupling constants (J) are reported in hertz. High-resolution mass spectra were obtained using a Thermo Fisher Q-Tof MicroTM instrument (Thermo Fisher Scientific, Waltham, MA, USA)

**N-Boc-1,4-butanediamine (2).** A solution of 1,4-diaminobutane (10.00 g, 113.44 mmol) in CH_2_Cl_2_ (200 mL) was treated with di-tert-butyl dicarbonate (Diboc, 4.95 g, 22.698 mmol) at 0 °C and stirred for 12 h. The mixture was filtered, and the filtrate was concentrated under reduced pressure. The residue was extracted with ethyl acetate (200 mL), washed with saturated NaCl solution, dried over anhydrous Na_2_SO_4_, and concentrated to afford compound 2 (3.89 g, 91%) as a pale yellow oil.

**3-Nitroquinolin-4-ol (4).** Nitric acid (31.51 g, 500 mmol) was added dropwise to a solution of 4-hydroxyquinoline (14.52 g, 100 mmol) in acetic acid (150 mL) at reflux. The reaction was stirred for 6 h. Upon completion, the mixture was filtered, washed with acetic acid and water, and dried at 65 °C to yield compound 4 (14.76 g, 93%) as a white solid. ^1^H NMR (400 MHz, DMSO-*d6*) δ 13.01 (s), 9.18 (s), 8.25 (dd, *J* = 8.2, 1.5 Hz), 7.79 (ddd, *J* = 8.4, 6.9, 1.5 Hz), 7.71 (dd, *J* = 8.4, 1.2 Hz), 7.51 (ddd, *J* = 8.2, 6.9, 1.2 Hz). ^13^C NMR (101 MHz, DMSO-*d*_6_) δ 167.70, 142.42, 138.29, 133.26, 131.01, 128.11, 126.01, 125.87, 119.52. HRMS (ESl): called for C_9_H_6_N_4_O_3_ [M + Na]^+^: 213.0276, found: 213.0258

**4-Chloro-3-nitroquinoline (5).** Thionyl chloride (SOCl_2_, 28.55 g, 240 mmol) and DMF (8.77 g, 120 mmol) were added to a solution of compound 4 (19.00 g, 100 mmol) in CH_2_Cl_2_ (200 mL) at reflux. The mixture was stirred for 12 h. After cooling, the reaction mixture was poured onto ice, and saturated sodium bicarbonate solution was added until gas evolution ceased. The mixture was extracted with dichloromethane. The organic layer was washed with saturated sodium bicarbonate, water, and brine, dried over anhydrous sodium sulfate, filtered, and concentrated to give the crude product (19.40 g, 93%), which was used directly in the next step.

**tert-Butyl (4-((3-nitroquinolin-4-yl)amino)butyl)carbamate (6).** A mixture of compound 5 (19.40 g, 93 mmol), N-Boc-1,4-butanediamine (17.51 g, 93 mmol), and K_2_CO_3_ (19.3 g, 120 mmol) in DMF (200 mL) was stirred at 80 °C for 4 h. The reaction was poured into water, and the precipitated solid was filtered and washed with ethanol to afford compound 6 (26.81 g, 80%) as a yellow solid. ^1^H NMR (400 MHz, CDCl_3_) δ 9.67 (s), 9.33 (s), 8.28 (d, *J* = 8.5 Hz), 7.96 (d, *J* = 8.3 Hz), 7.74 (t, *J* = 7.0 Hz), 7.47 (t, *J* = 7.1 Hz), 4.70 (s), 4.00–3.93 (m, 2H), 3.19 (q, *J* = 6.7 Hz, 2H), 1.94–1.80 (m, 2H), 1.67 (p, *J* = 7.1 Hz, 2H), 1.42 (s, 9H). ^13^C NMR (101 MHz, CDCl_3_) δ 156.14, 151.05, 150.63, 147.56, 132.79, 130.49, 127.08, 125.90, 125.55, 119.42, 49.16, 39.98, 28.50, 28.41, 27.53. HRMS (ESl): called for C_18_H_24_N_4_O_4_ [M + Na]^+^: 383.1690, found: 383.1667.

**tert-Butyl (4-((3-aminoquinolin-4-yl)amino)butyl)carbamate (7).** Compound 6 (5.00 g, 13.87 mmol) and palladium on activated charcoal (10% Pd, 500 mg, 1% *w*/*w*) were suspended in THF (70 mL). The reaction was stirred under a hydrogen atmosphere for 4 h. The mixture was filtered through a Celite pad, and the filtrate was concentrated under reduced pressure to yield compound 7 (4.35 g, 95%) as a thick solid, used directly in the next step.

**tert-Butyl (4-(2-butyl-1H-imidazo[4,5-c]quinolin-1-yl)butyl)carbamate (8)**. Trimethyl orthovalerate (1.47 g, 9.08 mmol) was added to a solution of compound 7 (2.00 g, 6.05 mmol) in toluene (30 mL) at reflux. The reaction was stirred for 4 h. The solvent was removed under reduced pressure, and the crude product was purified by column chromatography (5–20% methanol/dichloromethane) to afford compound 8 (1.80 g, 75%) as a white solid. ^1^H NMR (400 MHz, DMSO-*d*_6_) δ 7.99 (d, *J* = 7.9 Hz), 7.61 (dd, *J* = 8.4, 1.3 Hz), 7.41 (ddd, *J* = 8.3, 6.9, 1.3 Hz), 7.30–7.20 (m), 6.83 (t, *J* = 5.8 Hz), 6.47 (s, 2H), 4.49 (t, *J* = 7.5 Hz, 2H), 3.00–2.85 (m, 4H), 1.94–1.68 (m, 5H), 1.58–1.50 (m, 2H), 1.45 (h, *J* = 7.4 Hz, 2H), 1.33 (s, 9H), 0.96 (t, *J* = 7.3 Hz, 3H). ^13^C NMR (101 MHz, DMSO-*d*_6_) δ 155.65, 152.98, 151.67, 144.65, 132.20, 126.41, 126.25, 126.21, 121.16, 119.93, 114.79, 77.40, 44.59, 29.72, 28.20, 27.28, 26.57, 26.15, 21.97, 13.82. HRMS (ESl): called for C_23_H_33_N_5_O_2_ [M + H]^+^: 412.2712, found: 412.2697.

**tert-Butyl(4-(4-amino-2-butyl-1H-imidazo[4,5-c]quinolin-1-yl)butyl)carbamate (9).** m-Chloroperbenzoic acid (m-CPBA, 1.3 g, 7.56 mmol) was added to a solution of compound 8 (1.5 g, 3.78 mmol) in CHCl_3_ (20 mL) at 50 °C. The mixture was stirred for 2 h. Subsequently, 20% aqueous ammonium hydroxide (19.85 g, 113.4 mmol) was added, and stirring continued at 50 °C for 1 h. After cooling to room temperature, p-toluenesulfonyl chloride was added, and the reaction was stirred for an additional 4 h. The mixture was diluted with CHCl_3_ (80 mL) and washed with aqueous bicarbonate and brine. The organic layer was dried with Na_2_SO_4_ and concentrated. The crude product was purified by silica gel chromatography (20% DCM/MeOH) to yield compound 9 (1.10 g, 71%). ^1^H NMR (400 MHz, DMSO-*d*_6_) δ 7.99 (d, *J* = 7.9 Hz), 7.61 (dd, *J* = 8.4, 1.3 Hz), 7.41 (ddd, *J* = 8.3, 6.9, 1.3 Hz), 7.30–7.20 (m), 6.83 (t, *J* = 5.8 Hz), 6.47 (s, 2H), 4.49 (t, *J* = 7.5 Hz, 2H), 3.00–2.85 (m, 4H), 1.94–1.68 (m, 5H), 1.58–1.50 (m, 2H), 1.45 (h, *J* = 7.4 Hz, 2H), 1.33 (s, 9H), 0.96 (t, *J* = 7.3 Hz, 3H). ^13^C NMR (101 MHz, DMSO-*d*_6_) δ 155.65, 152.98, 151.67, 144.65, 132.20, 126.41, 126.25, 126.21, 121.16, 119.93, 114.79, 77.40, 44.59, 29.72, 28.20, 27.28, 26.57, 26.15, 21.97, 13.82. HRMS (ESl): called for C_23_H_33_N_5_O_2_ [M + H]^+^: 412.2712, found: 412.2697.

**1-(4-aminobutyl)-2-Butyl-1H-imidazo[4,5-c]quinolin-4-amine (10).** Compound 9 (1.35 g, 3.28 mmol) was dissolved in THF (20 mL) and stirred under a hydrogen chloride atmosphere for 4 h. The mixture was filtered to afford compound 10 (1.06 g, 93%). ^1^H NMR (400 MHz, D_2_O) δ 7.73 (d, *J* = 7.7 Hz), 7.51 (t, *J* = 7.4 Hz), 7.43 (t, *J* = 7.1 Hz), 7.30 (d, *J* = 7.2 Hz), 4.25 (t, *J* = 6.5 Hz, 2H), 2.93 (s, 3H), 2.84 (t, *J* = 7.8 Hz, 2H), 1.74 (tt, *J* = 8.4, 4.1 Hz, 6H), 1.44 (h, *J* = 7.4 Hz, 2H), 0.95 (t, *J* = 7.4 Hz, 3H). ^13^C NMR (101 MHz, D_2_O) δ 157.33, 146.72, 134.76, 132.69, 130.06, 125.85, 121.72, 120.77, 118.04, 111.42, 45.16, 38.83, 28.29, 26.07, 26.05, 23.73, 21.77, 13.04 C_18_H_25_N_5_ [M + H]^+^: 312.2188, found: 312.2174.

### 2.2. Biological Evaluation

#### 2.2.1. Reagents and Antibodies

TRIzol Reagent (D9108B) and SYBR Premix Ex Taq (RR420A) were purchased from TaKaRa (Kyoto, Japan). The anti-PRV-gB antibody was developed in-house. Anti-GFP (50430-2-AP) and anti-TLR7 (17232-1-AP) antibodies were purchased from Proteintech (Wuhan, China). HRP-conjugated goat anti-rabbit IgG (A16104) and goat anti-mouse IgG (PA1-74421) were obtained from Thermo Fisher Scientific (Waltham, MA, USA).

#### 2.2.2. Cells and Virus

PK-15 and THP-1 cells were maintained as monolayers at 37 °C with 5% CO_2_. PK-15 cells were cultured in DMEM (GIBCO), while THP-1 cells were cultured in RPMI 1640 (GIBCO, Waltham, MA, USA) supplemented with 10% FBS (GIBCO, Waltham, MA, USA), 100 units/mL penicillin, and 100 μg/mL streptomycin sulfate (Sangon, Shanghai, China). PRV-GFP (Recombinant PRV containing the GFP reporter gene) was gift from Han-zhong Wang (Wuhan institute of virology, China). VSV-GFP and PRV-QXX were gifts from Yong-Tao Li (Henan Agricultural University, China).

#### 2.2.3. Cell Viability Analysis

Cell viability was assessed using the CCK-8 assay (DingGuo, Beijing, China). PK-15 and THP-1 cells (1 × 10^4^ cells/well) were seeded into 96-well plates and treated with AXC-715 (0–50 μM) or DMSO for 24 h. CCK-8 reagent (10 μL) was added to each well, and plates were incubated at 37 °C for 3 h. Absorbance was measured at 450 nm using a Varioskan Flash microplate reader (Thermo Fisher Scientific, Waltham, MA, USA).

#### 2.2.4. Luciferase Reporter Assay

THP-1 Lucia NF-κB cells (WT and p65 KO) were seeded in 96-well plates (1 × 10^6^ cells/well) and treated with AXC-715 (0–50 μM) for 24 h. For luminescence measurement, 20 μL of cell suspension was mixed with 180 μL QUANTI-Luc (Invivogen, San Diego, CA, USA), and luminescence was recorded immediately.

#### 2.2.5. Molecular Docking

The crystal structure of hTLR7 (PDB ID: 5GMH) was prepared in PyMOL 3.1.0 (New York, NY, USA) by removing water molecules and non-relevant ligands (NAG, SO_4_). The native ligand RX8 was retained to define the binding pocket. AXC-715 was designed in ChemBioDraw 14.0.0.117 (Waltham, MA, USA) and saved in .sdf format. Protein and ligand were imported into Discovery Studio 19.1.0 (San Diego, CA, USA for preparation: hydrogen atoms were added, charges were calculated, force field parameters were assigned, and energy minimization was performed. Rotatable bonds were defined for the ligand. The binding site sphere (radius: 6.53686 Å) was centered on the RX8 coordinates (X = −15.0035, Y = −28.3855, Z = −12.0789). After removing RX8, molecular docking was performed, and results were visualized in PyMOL 3.1.0 (New York, NY, USA).

#### 2.2.6. Flow Cytometry

PK-15 cells were seeded into 24-well plates at a density of 1 × 10^5^ cells/mL. When the cells reached approximately 50% confluence, they were pretreated with AXC-715 at concentrations ranging from 0 to 20 μM for 4 h. The cells were infected with PRV-GFP at an MOI of 0.1 or VSV-GFP at an MOI of 0.1 for 1 h. After infection, the medium was replaced with fresh DMEM containing 1% fetal bovine serum (FBS) and AXC-715 at the corresponding concentrations, and the cells were incubated for an additional 24 h. GFP fluorescence in infected cells was observed using an inverted fluorescence microscope (OLYMPUS, Tokyo, Japan), and the percentage of GFP-positive cells was quantified by flow cytometry on a CytoFLEX instrument (BECKMAN COULTER, Miami, FL, USA) with data analyzed using CytExpert 2.5.0.77 (Beckman Coulter, Miami, FL, USA).

#### 2.2.7. RNA Extraction and Real-Time Quantitative PCR (RT-qPCR)

Total RNA was extracted from cell samples using TRIzol Reagent (9108, TaKaRa, Kyoto, Japan), and reverse transcription into cDNA was performed using the PrimeScript RT Reagent Kit (RR047A, TaKaRa, Kyoto, Japan). Quantitative real-time PCR (qRT-PCR) was conducted in triplicate using SYBR Premix Ex Taq (RR820A, TaKaRa, Kyoto, Japan). Gene expression levels were normalized to ACTB (β-actin), and relative quantification was calculated using the 2^−ΔΔCt^ method. The primer sequences used for qRT-PCR are presented in Table 1.

#### 2.2.8. Western Blot Analysis

Cells were lysed in a buffer containing 50 mM Tris-HCl (pH 8.0), 150 mM NaCl, 1% Triton X-100, 1% sodium deoxycholate, 0.1% SDS, and 2 mM MgCl_2_, supplemented with protease and phosphatase inhibitor cocktails (HY-K0010, HY-K0022; MedChemExpress, Monmouth Junction, NJ, USA). Protein concentrations were determined using a BCA Protein Assay Kit (BCA01, DingGuo, Beijing, China). Equal amounts of protein were separated by SDS-PAGE and transferred onto PVDF membranes (ISEQ00010, Millipore, Billerica, MA, USA). Membranes were blocked with 5% nonfat milk (A600669, Sangon, Shanghai, China) at room temperature for 1 h, followed by overnight incubation at 4 °C with primary antibodies against PRV gB, GFP, TLR7, and β-actin. After washing, membranes were incubated with horseradish peroxidase-conjugated secondary antibodies (goat anti-mouse IgG and goat anti-rabbit IgG) for 1 h at room temperature. Protein bands were visualized using Luminata Crescendo Western HRP Substrate (WBLUR0500, Millipore, Billerica, MA, USA) and detected with a GE AI600 imaging system.

#### 2.2.9. TCID_50_ Assay

PK-15 cells or TLR7^−/−^ PK-15 were seeded into 96-well plates at a density of 1 × 10^4^ cells/well. After 24 h, cells were infected with serially diluted virus (10^−1^ to 10^−12^) and incubated at 37 °C for 1 h. Unbound virus was removed by washing with PBS, and 100 μL of maintenance medium (DMEM supplemented with 1% fetal bovine serum) was added to each well. Cells were cultured for 3–5 days, and cytopathic effects (CPEs) were monitored daily. The 50% tissue culture infectious dose (TCID_50_) was calculated using the Reed–Muench method.

#### 2.2.10. Viral Attachment Assay

PK-15 cells were seeded in 6-well plates (1 × 10^5^ cells/well). At approximately 50% confluence, the medium was replaced with viral inoculum containing 10 μM AXC-715 and PRV-QXX (MOI = 1). Cells were incubated at 4 °C for 1 h to facilitate viral adsorption. Following adsorption, cells were washed three times with ice-cold PBS. Total RNA was extracted, reverse transcribed into cDNA, and the mRNA expression level of the PRV gB gene was quantified by qRT-PCR.

#### 2.2.11. Viral Entry Assay

Cells were inoculated with pre-chilled PRV-QXX (MOI = 1) and incubated at 4 °C for 1 h to allow viral adsorption. After washing three times with ice-cold PBS, cells were cultured in fresh medium containing 10 μM AXC-715 at 37 °C for 1 h to permit entry. Residual viral particles on the plasma membrane were removed by trypsin treatment (1 mg/mL). Total RNA was extracted, and viral genome copy number was quantified by qRT-PCR.

#### 2.2.12. Viral Replication Assay

Cells were infected with PRV-GFP (MOI = 0.1) at 37 °C for 1 h. The inoculum was replaced with fresh medium containing 10 μM AXC-715. Viral replication kinetics were monitored at 0, 6, 12, 24, and 36 h post-infection.

#### 2.2.13. Viral Assembly Assay

Cells were infected with PRV-QXX (MOI = 10) at 37 °C for 1 h. The inoculum was replaced with fresh medium containing 10 μM AXC-715. After 24 h, the efficiency of viral assembly in the supernatant was evaluated by determining the infectious titer (TCID_50_/mL) and quantifying the PRV genomic copy number.

#### 2.2.14. Viral Secretion Assay

Cells were infected with PRV-QXX (MOI = 10) at 37 °C for 1 h. The inoculum was replaced with fresh medium containing 10 μM AXC-715. At 24 h post-infection, samples were collected for viral titer determination. The ratio of intracellular to extracellular infectious particles relative to the total infectious titer was calculated to evaluate viral release efficiency.

#### 2.2.15. Statistical Analysis

All data were analyzed using Prism 8 software (GraphPad Software) and are presented as mean ± SEM. Each experiment was performed in triplicate (n = 3). Differences between groups were assessed using two-tailed Student’s t-tests. Statistical significance relative to the corresponding controls was indicated as follows: * *p* < 0.05, ** *p* < 0.01, and *** *p* < 0.001.

## 3. Results

### 3.1. Chemistry

**Scheme 1:**
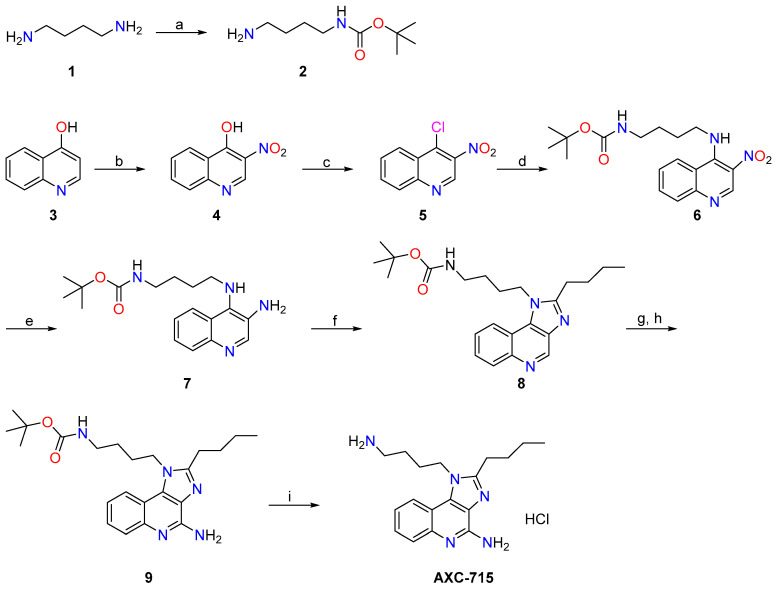
Synthesis of AXC-715. Reagent and conditions: (**a**) Diboc, CH_2_Cl_2_, 0 °C, 12 h; (**b**) HNO_3_, CH_3_COOH, reflux, 6 h; (**c**) SOCl_2_, CH_2_Cl_2_, DMF, reflux,12 h; (**d**) N-boc-1,4-butanediamine, K_2_CO_3_, DMF, 80 °C, 4 h; (**e**) Pd/C, H_2_, THF, 12 h; (**f**) trimethyl orthovalerate, Toluene, reflux, 4 h; (**g**) m-CPBA, CHCl_3_, 50 °C, 2 h; (**h**) NH_4_OH, p-toluenesulfonyl chloride, CHCl_3_, rt,4 h; (**i**) dry HCl gas, THF, ice-water 2 h.

The synthetic route for AXC-715 is illustrated in Scheme 1. The synthesis commenced with commercially available 1,4-diaminobutane (1) and 4-hydroxyquinoline (3). Compound 1 was mono-protected via reaction with di-tert-butyl dicarbonate (Boc_2_O) to yield intermediate 2. In parallel, 4-hydroxyquinoline was nitrated using nitric acid to afford the nitro-substituted derivative. Subsequent chlorination of the 4-hydroxyl group with thionyl chloride in dichloromethane yielded intermediate 4. Nucleophilic substitution of the chlorine in 4 with amine 2 furnished compound 6. Reduction of the nitro group in 6 under a hydrogen atmosphere generated amine 7, which underwent intramolecular cyclization with trimethyl orthovalerate to form the imidazoquinoline core 8. Oxidation of 8 with m-chloroperbenzoic acid (m-CPBA), followed by treatment with aqueous ammonia, produced compound 9. Finally, deprotection of 9 with dry hydrogen chloride gas in tetrahydrofuran (THF) yielded the target compound AXC-715 as a hydrochloride salt.

### 3.2. AXC-715 Promotes and Stabilizes TLR7 Dimer Formation

**Figure 1:**
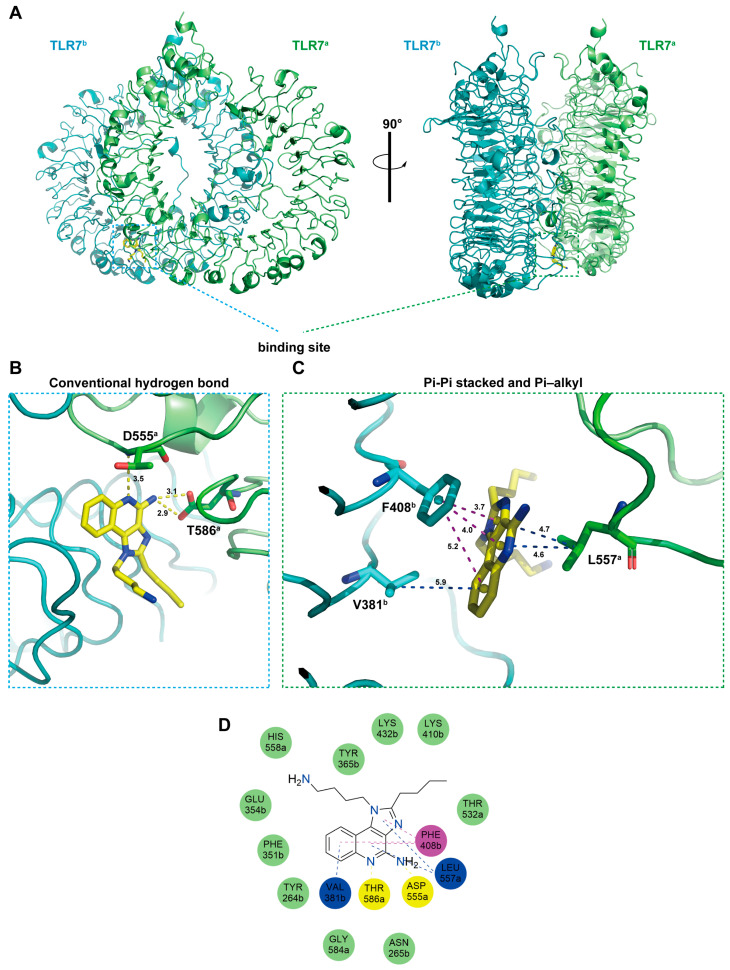
AXC-715 exhibits direct binding affinity to TLR7 protein. (**A**) Predicted binding model of the 2.2 Å cocrystal structure of the **AXC-715**–hTLR7 complex (PDB ID: 5GMH). TLR7 and its dimerization partner are colored green and cyan respectively, and **AXC-715** is shown in yellow. (**B**) Magnified view of the binding pocket highlighting key hydrogen bond interactions. (**C**) Close-up of the binding pocket illustrating π–π stacking and π–alkyl interactions. (**D**) Schematic representation of interactions between **AXC-715** and TLR7.

The binding mode of AXC-715 within the hTLR7 structure (PDB ID: 5GMH) was investigated to elucidate the mechanism of receptor activation. AXC-715 occupies the interface between TLR7 monomers, inserting into the binding pocket formed by the two protomers, thereby promoting and stabilizing the active dimer (Figure 1A). Molecular docking simulations indicate that AXC-715 forms specific hydrogen bonds with the side chains of D555 and T586 on monomer A. The pyridine nitrogen acts as a hydrogen bond acceptor for D555, while the amino group interacts with the hydroxyl of T586 (Figure 1B,D). Furthermore, the phenyl, pyridine, and imidazole rings of AXC-715 engage in π-π stacking with F408 of monomer B. The phenyl ring participates in a π-alkyl interaction with V381 (monomer B), while the pyridine and imidazole rings interact with L557 (monomer A) via π-alkyl forces (Figure 1C,D). By occupying this pocket, AXC-715 enhances ligand-protein affinity and locks the dimer in its active conformation. The combined hydrogen bonding and hydrophobic interactions stabilize the dimer interface, preventing dissociation and facilitating downstream signal transduction.

### 3.3. AXC-715 Induces Activation of NF-κB Response Element

**Figure 2:**
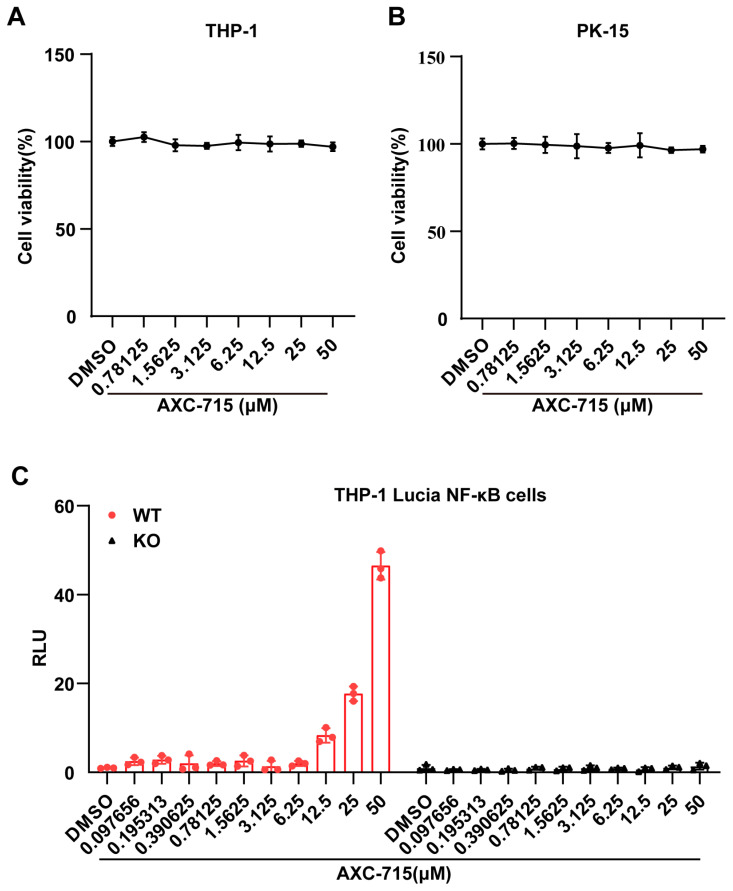
AXC-715 induces NF-κB activation in vitro. (**A**,**B**) THP-1 cells (**A**) and PK-15 cells (**B**) were treated with AXC-715 (0–50 μM) for 24 h. Cell viability was assessed using the CCK-8 assay. (**C**) THP-1 Lucia NF-κB (WT and p65 KO) cell lines were treated with the **AXC-715** for 24 h, then the cell-based activities of the selected compounds were quantified by normalizing compounds-induced reporter activity to DMSO-treated reporter activity. Data are mean ± SEM; n = 3. * *p* < 0.05, ** *p* < 0.01, *** *p* < 0.001.

The cytotoxicity of AXC-715 was assessed in PK-15 and THP-1 cells using the CCK-8 assay. Cells were treated with AXC-715 at concentrations ranging from 0 to 50 μM. As shown in Figure 2A,B, AXC-715 did not significantly reduce cell viability in either line, indicating a non-toxic profile at the tested concentrations. Subsequent evaluation of NF-κB pathway activation using THP-1 Lucia NF-κB reporter cells demonstrated a dose-dependent increase in promoter activity in wild-type cells (Figure 2C). This induction was absent in p65-knockout cells, confirming that AXC-715 activates NF-κB signaling via a p65-dependent mechanism.

### 3.4. AXC-715 Inhibits PRV Replication In Vitro

**Figure 3:**
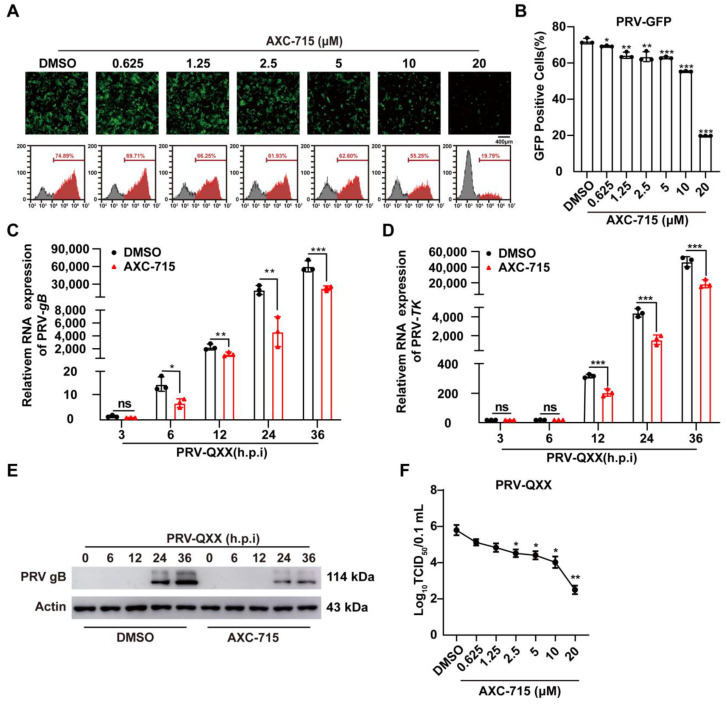
AXC-715 inhibits PRV replication in vitro. (**A**,**B**) PK-15 cells were infected with PRV-GFP and treated with **AXC-715** for 24 h. GFP expression was visualized by fluorescence microscopy and quantified by flow cytometry. (**C**–**E**) PK-15 cells were infected with PRV-QXX and then treated with **AXC-715** (10 μM). The mRNA expression levels of PRV gB and TK were analyzed by real-time quantitative PCR (RT-qPCR), and the protein expression level of PRV gB was assessed by Western blot analysis. (**F**) The effect of **AXC-715** on progeny virus production of PRV-QXX was evaluated by viral titration assay. Data are mean ± SEM; n = 3. ns *p* > 0.05, * *p* < 0.05, ** *p* < 0.01, *** *p* < 0.001.

The antiviral efficacy of AXC-715 against PRV was evaluated using a recombinant PRV-GFP strain. Fluorescence microscopy and flow cytometry showed a dose-dependent reduction in the GFP signal upon AXC-715 treatment, indicating inhibition of PRV proliferation (Figure 3A,B). qRT-PCR analysis confirmed a significant downregulation of PRV gB and TK gene transcription (Figure 3C,D). Western blot analysis further revealed decreased PRV-gB protein levels (Figure 3E). Viral titer assays (TCID_50_) corroborated these results, showing reduced production of progeny PRV-QXX (Figure 3F). These data demonstrate the potent anti-PRV activity of AXC-715.

### 3.5. AXC-715 Inhibits VSV Replication In Vitro

**Figure 4:**
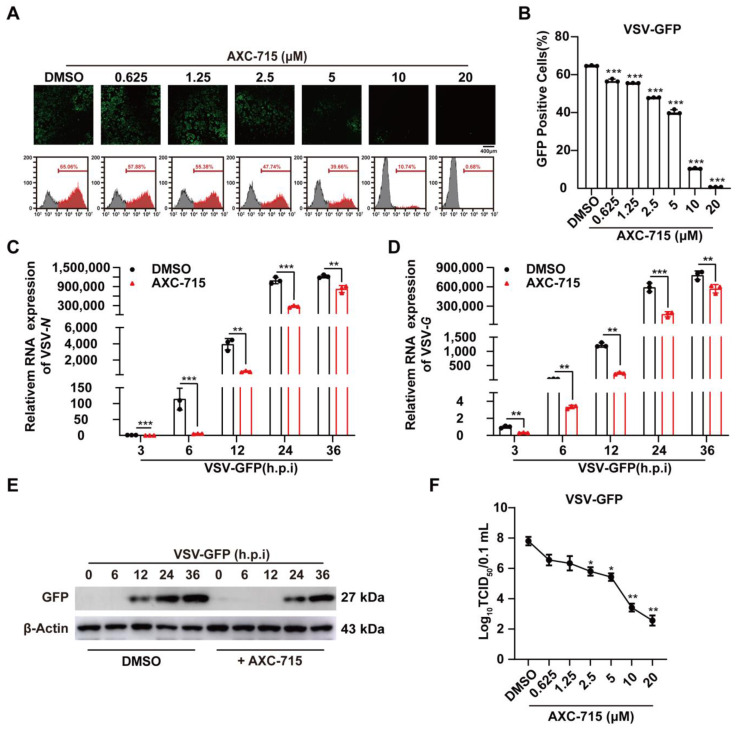
AXC-715 inhibits VSV replication in vitro. (**A**,**B**) PK-15 cells were infected with VSV-GFP and treated with **AXC-715** for 24 h. GFP expression was visualized by fluorescence microscopy and quantified by flow cytometry. (**C**–**E**) PK-15 cells were infected with VSV-GFP and then treated with **AXC-715** (10 μM). The mRNA expression levels of VSV-GFP N and G were analyzed by quantitative real-time PCR (qRT-PCR), and the protein expression level of PRV gB was assessed by Western blot analysis. (**F**) The effect of **AXC-715** on progeny virus production of VSV-GFP was evaluated by viral titration assay. Data are mean ± SEM; n = 3. * *p* < 0.05, ** *p* < 0.01, *** *p* < 0.001.

To assess broad-spectrum activity, the effect of AXC-715 on VSV was examined using VSV-GFP. Both fluorescence microscopy and flow cytometry indicated a dose-dependent decrease in GFP intensity, suggesting suppression of VSV replication (Figure 4A,B). qRT-PCR results showed significant inhibition of VSV N and G gene transcription (Figure 4C,D). Consistent with these findings, Western blotting detected reduced GFP protein expression (Figure 4E), and viral titer assays confirmed a decrease in progeny VSV-GFP production (Figure 4F). AXC-715 thus exhibits potent antiviral activity against VSV.

### 3.6. AXC-715 Inhibits Viral Replication

**Figure 5:**
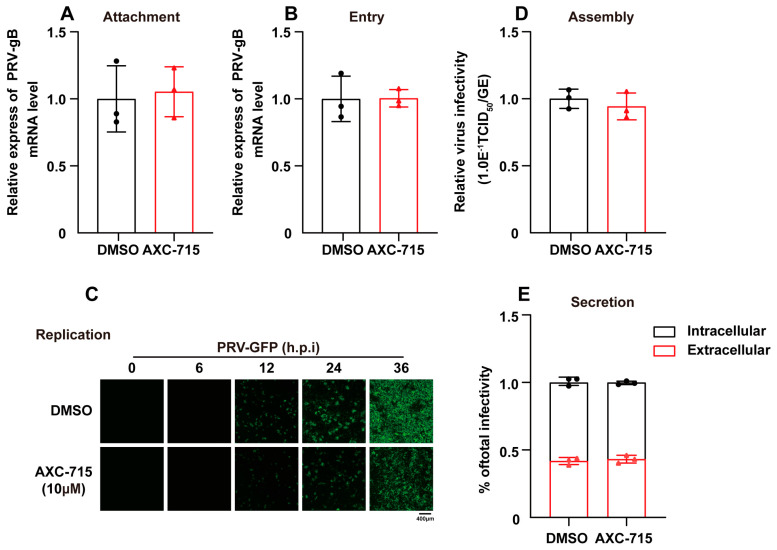
Study on the effect of AXC-715 on the life cycle of PRV. (**A**,**B**) Real-time PCR analysis of the effect of **AXC-715** (10 µM) on the adsorption and entry phases of PRV-QXX in PK-15 cells. (**C**) Immunofluorescence assay (IFA) assessing the impact of **AXC-715** (10 µM) on PRV-QXX replication in PK-15 cells. (**D**) Evaluation of **AXC-715** (10 µM) on the assembly phase of PRV-QXX in PK-15 cells by analyzing the ratio of infectious viral titers to viral genome copy numbers. (**E**) Analysis of the effect of **AXC-715** (10 µM) on the release phase of PRV-QXX in PK-15 cells by the ratio of viral titers in the supernatant to those inside the cells.

Mechanistic studies were performed to identify the stage of the viral life cycle targeted by AXC-715. First, attachment assays showed that co-incubation of PRV-QXX with AXC-715 at 4 °C did not alter gB mRNA levels compared to the control, indicating no effect on viral adsorption (Figure 5A). Next, entry assays were conducted; pre-adsorbed virus was allowed to enter in the presence of AXC-715 at 37 °C. qRT-PCR analysis revealed no significant difference in viral entry (Figure 5B). In contrast, replication assays monitoring GFP fluorescence over time showed a marked reduction in the signal upon AXC-715 treatment (Figure 5C). Additionally, AXC-715 did not interfere with viral assembly or release. These results suggest that AXC-715 specifically impairs the intracellular replication phase of PRV.

### 3.7. AXC-715 Targets TLR7 to Inhibit Viral Replication

**Figure 6:**
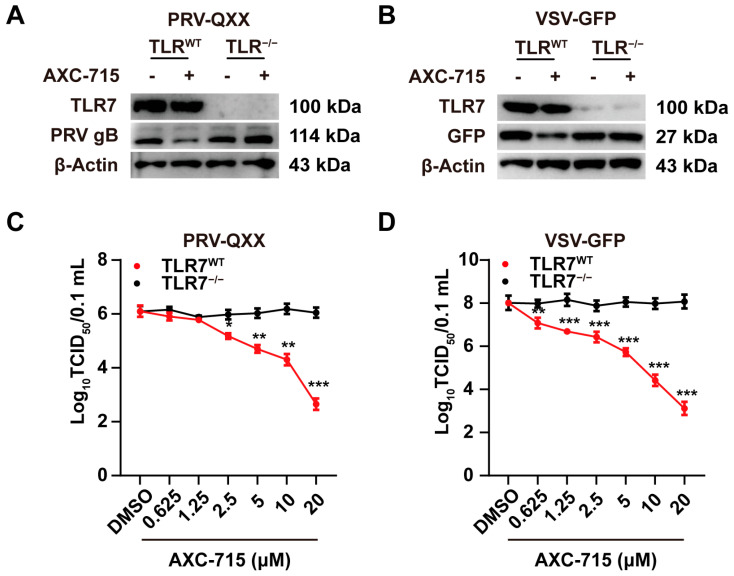
AXC-715 suppresses viral replication in a strictly TLR7-dependent manner. (**A**) Western blot analysis of PRV gB protein expression in TLR7^WT^ and TLR7^−/−^ PK-15 cells infected with PRV-QXX following AXC-715 treatment. (**B**) Western blot analysis of GFP protein expression in TLR7^WT^ and TLR7^−/−^ PK-15 cells infected with VSV-GFP following AXC-715 treatment. (**C**) The effect of AXC-715 on the production of PRV-QXX progeny virus in TLR7^WT^ and TLR7^−/−^ cells was evaluated by viral titration assay. (**D**) The effect of AXC-715 on the production of VSV-GFP progeny virus in TLR7^WT^ and TLR7^−/−^ cells was evaluated by viral titration assay. Data are mean ± SEM; n = 3. * *p* < 0.05, ** *p* < 0.01, *** *p* < 0.001.

To verify TLR7 dependence, TLR7 wild-type (TLR7^WT^) and TLR7 knockout (TLR7^−/−^) PK-15 cells were treated with AXC-715 and infected with PRV-QXX or VSV-GFP. Western blot analysis of PRV-gB and GFP protein expression revealed that AXC-715 significantly suppressed viral replication in TLR7^WT^ cells (Figure 6A,B). In contrast, no antiviral effect was observed in TLR7^−/−^ cells. This confirms that the antiviral activity of AXC-715 is strictly mediated through the TLR7 signaling pathway.

## 4. Discussion

This study elucidates the structural and functional mechanisms underlying the antiviral activity of AXC-715, a novel small-molecule agonist of TLR7. We demonstrate that AXC-715 functions as a potent molecular stabilizer of TLR7. Structural analysis of the AXC-715–hTLR7 complex revealed that the ligand occupies a critical position at the dimer interface, engaging key residues—D555 and T586 via hydrogen bonding, and F408, V381, and L557 through π-π and π-alkyl interactions. This binding mode facilitates the formation of the active “m”-shaped dimer, bringing the C-termini of the receptor into close proximity to recruit downstream adaptor proteins [27,28]. Unlike endogenous ligands such as guanosine or ssRNA, which often require specific coordination within the binding pocket [29], AXC-715 acts as a “molecular glue,” effectively locking the receptor in an active conformation through direct intermolecular contacts. This stabilization likely lowers the energy threshold for dimerization and sustains signaling output, accounting for the robust activation of NF-κB observed in our experiments.

Functionally, the capacity of AXC-715 to inhibit the replication of both DNA (PRV) and RNA (VSV) viruses underscores the potential of TLR7 agonists as broad-spectrum, host-directed antiviral therapeutics. Time-of-addition assays indicated that AXC-715 specifically impairs viral replication without affecting viral adsorption, entry, assembly, or release. This phenotype suggests that the antiviral effect is not due to direct virucidal activity but is mediated by the induction of a cellular antiviral state. Upon TLR7 activation, the NF-κB and IRF7 pathways are triggered, leading to the upregulation of IFNs and ISGs [30]. These ISGs establish an intracellular environment restrictive to viral replication. The complete abolition of antiviral activity in TLR7-knockout cells confirms the on-target specificity of AXC-715 and highlights its role as a host-directed therapeutic agent capable of circumventing viral resistance mechanisms that often plague direct-acting antivirals [31,32].

Beyond its immediate antiviral efficacy, AXC-715 provides critical insights into the pharmacological regulation of TLR7. The ability of small-molecule ligands to promote dimer formation offers a strategy to fine-tune immune responses. By stabilizing the active dimer, AXC-715 may enhance receptor sensitivity to low-level viral PAMPs that might otherwise evade detection [33]. This sensitization effect could be particularly beneficial during early infection or in cases of immune evasion. However, the potent activation of TLR7 necessitates a careful evaluation of the therapeutic window, as chronic or excessive signaling has been associated with immunopathology, including autoimmune disorders and cytokine storms [34]. While our in vitro studies showed no detectable cytotoxicity in PK-15 or THP-1 cells, the in vivo safety profile remains to be determined. Future studies must focus on the pharmacokinetics and biodistribution of AXC-715 to ensure that sustained receptor activation does not lead to excessive inflammation or tissue damage.

In conclusion, AXC-715 exemplifies a structure-guided, receptor-targeted strategy that leverages TLR7 dimer stabilization to modulate innate immunity. Its dual capacity to enhance NF-κB signaling and inhibit viral replication across distinct viral families highlights its potential as a versatile immunomodulatory agent. While alternative antiviral strategies, including immunomodulators, interferons, and antioxidants, have been explored, our study specifically focused on the host-directed approach using the TLR7 agonist AXC-715. This strategy activates innate immunity to establish a broad-spectrum antiviral state, potentially overcoming virus-specific resistance mechanisms commonly observed with direct-acting antivirals. Future studies could explore combinatorial approaches, assessing the synergy between TLR7 agonists and other biological response modifiers to further enhance antiviral efficacy and minimize potential side effects. These findings provide a valuable framework for the rational design of next-generation TLR7 agonists, aiming to optimize the balance between potent antiviral efficacy and acceptable safety profiles for clinical application.

## Figures and Tables

**Table 1:** Primers used for gene cloning and RT-qPCR analysis.

Name	Sequence (5′-3′)	Product Size (bp)
Q-Sus-β-actin-F	CTGAACCCCAAAGCCAACCGT	317
Q-Sus-β-actin-R	TTCTCCTTGATGTCCCGCACG	
PRV-gB-F	GGCATCGCCAACTTCTTCC	237
PRV-gB-R	CCTCGTCCACGTCGTCCTC	
PRV-TK-F	GGCGTACTGGCGCACTCTG	279
PRV-TK-R	ATGTCCCCGACGATGAAGC	
VSV-N-F	TGATAGTACCGGAGGATTGACGAC	127
VSV-N-R	CCTTGCAGTGACATGACTGCTCTT	
VSV-G-F	TTATTGCCCGTCAAGCTCAG	211
VSV-G-R	GCATTGTTCTACAGATGGA	

## Data Availability

The data that support the findings of this study are available from the corresponding author upon request.
